# Infant and young child feeding practices are associated with childhood anaemia and stunting in sub-Saharan Africa

**DOI:** 10.1186/s40795-022-00667-9

**Published:** 2023-01-10

**Authors:** Aaron Kobina Christian, Eric Afful-Dadzie, Grace S. Marquis

**Affiliations:** 1 Regional Institute for Population Studies, University of Ghana, Legon-Accra, Accra, Ghana; 2 Department of Operations and Management Information Systems, University of Ghana Business School, Accra, Ghana; 3 School of Human Nutrition, McGill University, Montreal, QC Canada

**Keywords:** Anaemia, Stunting, Co-occurrence of anaemia and stunting: feeding practice, Decision tree

## Abstract

**Background:**

The co-occurrence of anaemia and stunting (CAS) presents acute development and morbidity challenges to children particularly in sub-Saharan Africa (SSA). Evidence on the effect of child feeding recommendations on CAS is scarce.

**Methods:**

We used data from 22 recent Demographic and Health Surveys in SSA countries to examine the association between caregivers’ implementation of recommendations on infant and young child feeding and the CAS in their 6- to 23-mo-old children.

**Results:**

Overall, in multiple logistic regression models, child feed index score, high wealth of household, increasing household size, household head with at least secondary school education, improved sanitation of household, an increase in caregiver’s age and caregiver’s with at least secondary education were associated with lower odds of CAS (i.e., AOR: 0.86; 95% CI; 0.84 – 0.88: 0.75; 0.69 – 0.82: 0.98, 0.98 – 0.99: 0.76, 0.70 – 0.83: 0.81, 0.74 – 0.87: 0.87, 0.81 – 0.94: 0.69, 0.62 – 0.77 respectively). Having a diarrhoea in the past 2 weeks and having fever in the past month were associated with higher odds of CAS (AOR:1.1, 95% CI; 1.0 – 1.2: 1.1, 1.0 – 1.2, respectively). Results from the decision tree analysis showed that the educational level of women was the most important predictor of CAS, followed by child feeding score, the level of education of the family head and state of drinking water.

**Conclusion:**

The results buttress the importance of interventions aimed at improving feeding practices and parental educational as a vehicle to improve children’s nutritional status.

**Supplementary Information:**

The online version contains supplementary material available at 10.1186/s40795-022-00667-9.

## Introduction

Anaemia is defined as having low haemoglobin concentration or red blood cell mass as resulting from either nutritional factors (e.g., deficiencies of iron, folic acid, vitamin B_12_ and vitamin A), infections (e.g., soil-transmitted helminths, malaria, tuberculosis and HIV/AIDS) and/or due to genetic factors (e.g., thalassemia) [1].The global prevalence of anaemia in 2010 was estimated at 32.9% with sub-Saharan Africa (SSA) among the hardest hit regions alongside South Asia [2]. According to the World Health Organization (WHO), an estimated 273.2 million children aged 6–59 months were anaemic in 2011 [3]. Stunting on the other hand is assessed by measuring child’s length or height and interpretating the measurement by comparing them with an acceptable set of standard values per the child’s sex and age category. Stunting is considered as the best indicator for assessing children’s well-being. Over the past two decades whereas global numbers of stunted children have decreased in most regions, SSA has witnessed an increase [4].

Child growth was captured as a key indicator of development by the Millennium Development Goals [5] and subsequently within the Sustainable Development Goals (SDGs) [6]. The SDG Target 2.2. aims to end all forms of malnutrition, including stunting in children. Conversely, childhood anaemia influences under five years mortality, delayed motor development, reduced mental capacity and poor educational attainment [7] and substantial economic burden [8, 9]. The consequence of stunting includes low educational performance, suboptimal function later in life and, for pregnant women, adverse effects such as restricted uterine blood flow and growth of the uterus, placenta, and foetus [10].

Most undernutrition problems share overlapping risk factors, especially with respect to their proximate determinants [15, 16]. Factors such as poor socioeconomic status, inadequate health service utilization, suboptimal childcare practices and poor water and sanitation often serve as correlates of multiple health conditions including malnutrition outcomes [11, 12]. Given the overlapping causes of anaemia and stunting, their co-occurrence would be expected to occur particularly in poor childcare settings [13].

There have been several adaptations of the oft-cited United Nations Children’s Fund conceptual framework that speaks to the correlates of childhood nutritional status. An example of such a framework is the WHO framework that gives importance to inadequate complimentary child feeding practices -poor quality food, low dietary diversity and intake of food, infrequent and inadequate feeding, and insufficient frequency of feeding[14, 15]. It is generally agreed that appropriate infant and young child feeding (IYCF) is likely to increase child survival and promote optimal growth and development and has the most impact on children between six months and two years. In 2002, the WHO and UNICEF adopted a global strategy for IYCF aimed at bringing global attention to the impact of feeding practices. The WHO has since introduced a set of indicators for complementary feeding among children aged 6 to 23 months to track and encourage optimal feeding practices [16].

Although research on the effect of single feeding practice on nutritional outcomes is critical, it does not permit the examination of child feeding practices on children’s health and nutrition outcome [17]. Apart from a study by Gebremedhin (2019), pooled indicators for IYCF practices across the entire SSA have not been published. Additionally, critically missing is the adequate scholarship examining the relationship between IYCF practices and childhood nutrition outcomes (including anaemia, stunting, and the co-occurrence of anaemia and stunting (CAS)), which was the primary reason for which the indicators were generated. The aim of the current study is to use the most recent nationally representative data across the SSA to examine the relationship between IYCF practices and CAS in 6- to 23-months-old children. The key research questions addressed in this analysis are (1) Does IYCF predict CAS and (2) what is the relative importance of the association of other covariates to CAS in sub-Saharan Africa?

## Methods

### Data sources and procedures

We used data from the most recent Demographic and Health Surveys (DHS) of selected sub-Saharan Africa countries (SSA) provided by the Integrated Public Use Microdata Series (IPUMS) (Minnesota Population Centre). The DHS employs a stratified two-stage cluster sampling technique to select census enumeration based on probability (proportional to area size), then a random selection of households from a listing of households within the selected enumeration area. These surveys collect household and individual information on a wide range of themes, using comparable questionnaires across study countries. For selected households, a member who is usually the household head answers general questions on household socioeconomic and demographic characteristics through a face-to face interview.

All consenting women within the reproductive age bracket (i.e., 15 to 49 years old) are also interviewed on questions such as their reproductive health, their children’s dietary intake and their child feeding practices. Caregivers also provide information on the index children (i.e., children between 6 to 59 months) in the selected households. Children’s length/height and weight, and haemoglobin concentration are also assessed. The surveys also gather information on the household members and characteristics of the household.

IPUMS DHS was established in 2012 to support comparative over-time and international research with the DHS [18]. IPUMS provides added value to nationally represented survey and census data by coding variables consistently across different countries and over time. IPUMS also provides a systems integration and documentation which enables researchers to easily merge information from different countries and across different data types [19]. Interested variables and samples (i.e., countries) were selected on IPUMS data request portal and submitted. The pooled data us then downloaded automatically. The following were the inclusion criteria for selecting surveys for the current analysis: (i) only sub-Saharan African countries, (ii) survey period between 2008 and 2017, and (iii) included children’s anaemia and stunting status. The DHS is a nationally representative cross-sectional household survey that is conducted approximately every 5 years. All DHS follow standardised procedures in the survey design and data collection across study countries [20].

### Ethical considerations

Ethical clearances for the DHS studies are sought from the Ethics Committee of ORC Macro Inc. and from the Institutional Review Boards and Ethics Boards of partner institutions (e.g., Ministries of Health) of the studied countries. Protocols adopted for the DHS ensure that standards for the protection of respondents’ privacy and confidentiality are strictly adhered to. The Inner-City Fund International ensures that the survey conforms with the regulations for the respect of human subjects as stipulated by the United States Department of Health and Human Services. All procedures were performed in accordance with guidelines in accordance with the ethical standards as laid down in the 1964 Declaration of Helsinki and its later amendments or comparable ethical standards. As this study used data from the DHS provided through IPUMS, no further ethical approval or consideration was required. Additional information concerning protocols and ethical standards of the DHS can be found at https://dhsprogram.com/methodology/Protecting-the-Privacy-of-DHS-Survey-Respondents.cfm ( accessed on 2^nd^ November 2022). Written informed consent was obtained from all study participants.

### Measures

#### Main outcome

Childhood anaemia was defined as having a haemoglobin (Hb) level of < 11.0 mmol/L in children 6 to 59 months old, based on WHO classification [3]. The length/height-for-age Z-scores (LAZ/HAZ) for children were calculated based on child sex, age, and length (if aged < 2 years) or height (if aged ≥ 2 years) using WHO Anthro software version 3.2 [21]. Children with a LAZ/HAZ less than –2 were considered stunted. Thus, CAS was considered as having both a Hb level < 11.0 mmol/L and LAZ/HAZ < -2.

#### Main independent variable-IYCF practice

An IYCF index was created for this study based on age-specific (6 to 8 months, 9 to 23 months) feeding recommendations (Table 1). Breastfeeding is recommended for at least 24 months [22]; 2 points were given for children in all age categories who were breastfed and 0 if not breastfed. Use of bottles are discouraged [23]; 1 point was given for not using a bottle and 0 if a bottle was used. Dietary diversity was evaluated based on the number of food groups a child ate in the past 24 h using a list of seven food groups: grains, roots and white tubers; legumes; nuts and seeds; dairy products; flesh foods (meat, poultry, fish, and offal); eggs; pro-vitamin A-rich foods (yellow and orange-fleshed roots and tubers, orange-fleshed fruits, and dark green leafy vegetables); and other fruits and vegetables [24]. A published scoring scheme for diet diversity was modified and used as part of a feeding index as follows: for all age categories, a score of 2 points for eating from 4 or more food groups, 1 point was awarded to those who ate from 1- 3 food groups, and 0 was assigned to children who did not eat from any food group over the past 24 h. Based on the recommended meal frequency, 6- to 8-mo-old and 9- to 23-mo-old children who were fed at least twice and thrice times, respectively, were given 2 points. One point was given for fewer meals and 0 for no meals. Taking cue from previous studies a final IYCF index was generated as a summation of the scores obtained for each variable described [25]. The feeding scores were categorized as ‘Low’ and ‘High’ based on computation of the 50^th^ percentile.

**Table 1:** Variables and scoring system used to create the child feeding index for 6 to 23 months old children, by age group

	Age category		
Variables	6 to 8 months	9 to 23 months	Rationale for scoring
Is breastfed	No = 0; Yes = 2	No = 0; Yes = 2	Breast milk provides at least half of the energy requirements for children 6 to 12 months and one third for those between 12 to 24 months. Reduces risk of acute disease and chronic diseases provides long-term coronary health (Brown et al., 1990; Kathyrn Dewey, 2008)
Uses bottle	No = 1; Yes = 0	No = 1; Yes = 0	Bottle feeding is linked to unhygienic conditions increasing the risk of illness and mortality (Boone et al., 2016; Muhammad et al., 2018)
For past 24 h			
Dietary diversity score^a^	0 = 0 1 to 3 = 1 ≥ 4 = 2	0 = 0 1 to 3 = 1 ≥ 4 = 2	Having a more diverse diet is a proxy for adequate micronutrient-dense foods. Consumption from at least 4 food groups over 24 h. is considered adequate and likely to contain at least one fruit or vegetable and one animal source food in addition to a staple food, usually cereals or other starchy staples (Working Group on Infant and Young Child Feeding Indicators, 2006)
Meal frequency	0 meals/d = 0 1 meal/d = 1 ≥ 2 meals/d = 2	0 meals/d = 0 1 to 2 meals/d = 1 ≥ 3 meals/d = 2	The required meal frequency for children is dependent on their energy requirement (Kathyrn Dewey, 2008)
Maximum score	7 points	7 points	

^a^Dietary diversity from 24-h recall; maximum score of 7 when a child consumes food items from all 7 food groups: grains and tubers, legumes, dairy, flesh (red meat, fish, offal), eggs, vitamin A rich fruits, fruits and vegetables

#### Other covariates

Selection of covariates was guided by the UNICEF framework for childhood malnutrition and the WHO framework that gives importance to inadequate complimentary child feeding practices [26] and other socio-demographic characteristics that known to have an influence on childhood malnutrition. Household characteristics such as wealth status, total number of people (i.e., household size), access to improved sanitation and drinking water were included [27]. The following were considered as improved water sources: piped water, boreholes, or tube wells, protected dug wells, protected springs, rainwater, and packaged or delivered water and the unimproved sources were unprotected dug well, unprotected spring, river, dam, lake, pond, stream, canal, and irrigation canal. Improved sanitation facilities were flush/pour flush to piped sewer systems, septic tanks, or pit latrines; ventilated improved pit latrines, composting toilets, or pit latrines with slabs with pit latrines without a slab or platform, hanging latrines or bucket latrines and open defecation considered as unimproved facilities. Child characteristics that were selected included having experienced diarrhoea and fever of a specified period [28]. Whether or not a child was given either vitamin A or iron supplements within the past 6 months was also assessed.

### Statistical analysis

Factoring in the sampling design of the DHS data, sampling weights were calculated to account for differential probabilities of selection and participation of households across countries. Weighted proportions and 95% confidence intervals (CI) were calculated with STATA version 14.2 (Stata, College Station, TX, USA). The *svyset* command in STATA was used to describe the sample design. The *svy*: prefix is used before commands for running models such as *regress* or *logistic*. Chi-square was used to test the association between child feeding index terciles (low, medium, and high) and CAS in a bivariate analysis. For individual countries, binary logistic regression was used to test whether the magnitude and statistical significance of the association between the child feeding index and CAS remained after controlling for other covariates noted to influence anaemia and stunting.

We further used decision tree analysis to comprehensively understand the weight of the factors that influenced CAS observed, that is to examine correlates with respect to the extent of their importance in predicting the outcome of interest. A decision tree is a non-parametric supervised learning method used for both classification and predictive analysis problems. The goal is to create a model that concurrently predicts and classifies the value of a dependent variable (in this case CAS) by learning simple decision rules inferred from the data attributes [29]. Visually, it is an inverted tree where each node represents a given data attribute, each branch a decision rule (condition) and each leaf represent an outcome (categorical or continuous value). In each node of the tree, a splitting rule (referred to as a condition) is applied to determine which class a datum should belong for a specific attribute that has a property of data associated with the node [30]. The algorithm has an added power of inferring the dominant attributes most important to the prediction. In this instance, the condition of the node which is closest to the root of the tree is considered the most influential or the most important as far as the prediction is concerned.

To obtain the tree and nodes, we employed the gain ratio methodology as the criterion on which attributes were selected for splitting. The gain ratio criterion is a variant of information gain used in measuring how well a given attribute separates the training examples based on their target classification. The goal for the decision tree analysis was to identify relatively important factors that predict CAS. Automatic feature selection was performed in the RapidMiner 2018 software (RapidMiner, Boston) to select only attributes that contributed significantly to the prediction of CAS. Criteria for selection included (1) attributes (variables and the categories of that variable) that had a strong relationship with the outcome variable (CAS) and (2) independent variables that had very low correlation between themselves.

## Results

### Characteristics of the study sample

The final analytic sample for assessing the association of child feeding index with CAS was 33,846 caregiver-child dyads across all the selected 22 SSA countries. The heads of the households were mostly men (79%), and only about 28% of the household heads had more than a secondary level of education. In terms of household conditions, slightly more than half of the households (56%) had improved water source, and 45% had improved toilet facilities. The mean household size was 7.2 (4.6 SD). Over half of the countries (12/22, 55%) had less than 20% of women attaining secondary or higher education. Fifteen out of the twenty-two countries had 20% or more of mothers indicating that their children had diarrhoea or fever over the two weeks prior to the survey. There was no vitamin A supplementation in Angola, no iron supplementation in Zimbabwe, South Africa, Rwanda, Namibia, and Lesotho. The household, caregiver, and child characteristics that may influence the nutritional outcome of the children varied widely across the countries (Table 2). The prevalence of households with improved water source and access to improved sanitation facility was lowest in Madagascar (31%) and highest in Malawi (80%). Both maternal and head of household education varied greatly across the SSA countries. Burkina Faso and Niger had the lowest and South Africa had the highest educational achievement.

**Table 2:** Sociodemographic characteristics and health and nutrition indicators for the study sample population, by country^a^

	Angola	Benin	Burkina Faso	Burundi	Cameron	Cote d'Ivoire	DRC	Ethiopia	Ghana	Guinea	Lesotho
Year of survey	2015	2011	2010	2016	2011	2011	2013	2016	2014	2012	2014
**Household**											
Household size (#)	6.2 ± 2.8	6.5 ± 3.2	7.5 ± 3.8	5.7 ± 2.1	7.9 ± 4.2	7.8 ± 4.5	6.8 ± 3.0	5.9 ± 2.1	5.5 ± 2.6	8.5 ± 4.5	6.0 ± 2.5
Household head											
Male (Yes)	71.2	85.5	91.3	79.2	85.9	84.4	78.6	87.0	75.8	85.5	70.8
Attained at least secondary education (Yes)	53.9	21.8	5.9	12.9	42.1	21.2	70.6	13.5	66.6	20.3	36.3
Access to improved water source (Yes)^b^	38.5	67.2	72.2	78.3	60.4	59.0	40.5	50.2	63.3	69.8	62.3
Access to improved sanitation (Yes)^c^	61.7	31.9	23.5	52.2	53.1	42.0	38.8	9.8	66.2	36.8	69.9
Wealth tertiles^d^											
Low	38.5	45.7	40.8	42.4	48.1	43.0	45.4	43.9	43.3	46.9	41.5
Middle	18.0	21.7	23.4	22.0	19.0	21.2	18.7	23.3	19.7	19.6	25.2
Highest	43.5	32.5	35.8	35.6	32.9	35.8	35.9	32.9	37.1	33.5	33.3
**Female caregiver**											
Age (y)											
15 to 24	41.7	24.6	33.2	25.6	39.7	37.7	32.1	27.9	25.2	35.3	51.5
24 to 49	58.3	75.4	66.8	74.4	60.3	62.3	67.9	72.1	74.8	64.7	48.6
Attained at least secondary education (Yes)	33.2	14.2	5.5	11.4	34.0	9.7	39.1	7.6	53.0	10.9	56.6
**Child**											
Had diarrhea in past 2 wk (Yes)	26.1	13.3	23.1	37.8	33.8	27.2	31.2	19.4	16.1	23.0	23.5
Had fever in past 2 wk (Yes)	20.1	16.3	30.5	47.9	35.3	34.6	37.7	20.1	15.5	37.5	19.9
Vitamin A in past 6 mo (Yes)	0.0	50.2	67.4	72.4	51.7	61.7	68.7	42.7	70.1	38.1	72.5
Iron supplement in past 7 d (Yes)	11.1	39.3	8.3	7.7	9.9	16.5	15.6	7.8	22.9	10.1	0.0
Anaemia (Yes)^e^	78.7	67.4	94.2	69.3	73.6	86.5	68.1	72.0	77.7	85.1	61.0
Stunted (Yes)^f^	37.6	37.8	30.6	52.9	27.5	27.2	34.0	32.9	14.6	22.5	29.2
CAS (Yes)^g^	29.5	25.0	29.1	36.8	21.3	23.8	22.6	24.1	12.0	19.9	20.8

	Madagascar	Malawi	Mali	Mozambique	Namibia	Niger	Rwanda	Senegal	South Africa	Tanzania	Zimbabwe
Year of survey	2008	2016	2012	2011	2013	2012	2014	2017	2016	2015	2015
**Household**											
Household size (#)	6.0 ± 2.5	5.3 ± 2.0	7.2 ± 3.5	5.8 ± 2.4	6.7 ± 3.3	7.6 ± 3.7	5.1 ± 1.8	14.3 ± 8.5	6.3 ± 3.0	7.2 ± 4.4	5.5 ± 2.4
Household head											
Male (Yes)	85.5	72.5	92.1	72.3	47.3	85.7	80.6	74.0	45.7	82.9	62.7
Attained at least secondary education (Yes)	22.7	35.7	10.5	19.3	62.5	6.8	11.3	17.1	86.0	18.2	75.6
Access to improved water source for drinking (Yes)^b^	31.3	80.0	57.8	40.1	71.0	63.1	65.1	46.2	60.9	47.0	64.4
Access to improved sanitation (Yes)^c^	5.3	81.0	37.4	19.6	35.9	19.0	69.6	66.4	68.9	69.4	58.1
Wealth tertiles^d^											
Low	49.1	44.9	48.3	44.0	47.3	47.6	36.9	46.4	47.0	45.4	47.4
Middle	19.4	20.5	19.9	18.4	19.6	21.4	21.9	19.3	20.7	18.8	17.7
Highest	31.5	34.7	31.8	37.6	33.1	31.0	41.2	34.3	32.3	35.8	34.9
**Female caregiver**											
Age (y)											
15 to 24	42.2	45.3	32.3	39.6	35.8	27.6	25.0	29.7	34.6	37.7	36.9
24 to 49	57.9	54.7	67.7	60.4	64.2	72.4	75.0	70.3	65.4	62.3	63.1
Attained at least secondary education (Yes)	20.6	23.1	9.1	12.0	72.2	6.4	13.7	18.1	90.0	17.3	65.7
**Child**											
Had diarrhoea in past 2 wk (Yes)	16.2	37.3	13.4	18.3	35.1	28.0	21.9	27.8	18.8	21.5	30.9
Had fever in past 2 wk (Yes)	14.5	36.9	13.6	17.2	33.2	22.8	25.1	28.5	26.1	22.6	17.5
Vitamin A in past 6 mo (Yes)	43.2	67.6	53.0	69.6	86.5	60.4	82.4	57.4	78.9	43.1	76.5
Iron supplement in past 7 d (Yes)	1.6	11.4	18.7	21.0	0.0	10.5	0.0	2.9	0.0	1.4	0.0
Anaemia (Yes)^e^	62.4	80.9	88.6	79.3	63.8	86.4	52.2	83.6	68.7	75.3	55.7
Stunted (Yes)^e^	48.2	32.9	30.5	41.5	17.9	38.9	37.0	16.3	33.3	32.2	26.4
CAS (Yes)^g^	31.2	27.3	27.5	34.3	11.3	33.6	18.6	14.4	25.7	24.6	14.9

*DRC* Democratic Republic of the Congo, *CAS* co-occurrence of anaemia and stunting

^a^Sample includes only households with complete data on children’s height, age, and haemoglobin level. Values represent mean ± standard deviation or percentage

^b^Improved water source: piped household water connection, public standpipe, borehole, protected dug well, protected spring and rainwater

^c^Improved sanitation: ventilated improved pit latrines and pit latrines with a slab or covered pit.

^d^Wealth quintiles were calculated from an asset-based wealth index using assigned asset weights from a principal components analysis to create standardized asset scores.

^e^Anemia:hemoglobin concentration of < 110 mmol/L.

^f^ Stunted: length/height-for-age Z-score < –2

^g^CAS: haemoglobin level < 11.0 mmol/L and length/height-for-age Z-score < -2

### Nutritional status of children and child feeding practices: Prevalence of anaemia, stunting and CAS

Overall, the prevalence of anaemia, stunting and the CAS among the sample children were 76%, 32% and 25%, respectively. More than half of the study countries had over 80% of mothers breastfeeding their 6- to 23-months-old children (Table 3). South Africa was the only country with less than half of mothers breastfeeding. In Burkina Faso, breastfeeding was close to universal (94%) and bottle feeding almost non-existent (1%) for this age group. Overall, only 16% of the children consumed from at least four food groups.

**Table 3:** Feeding practices reported by caregivers, by country^a^

	Angola	Benin	Burkina Faso	Burundi	Cameron	Cote d'Ivoire	DRC	Ethiopia	Ghana	Guinea	Lesotho
Currently...											
Breastfeeding	77.6	72.9	93.7	91.8	67.7	75.1	87.4	88.3	83.2	89.1	64.4
Uses bottle	15.8	14.9	1.3	11.5	7.7	4.5	5.6	14.8	12.1	4.9	22.5
**Dietary diversity for past 24 h**											
Consumed from no food group	16.2	23.5	28.4	13.2	10.7	16.0	19.7	24.3	11.6	24.7	8.2
Consumed from 1—3 food groups	57.8	52.0	68.7	75.4	67.2	74.3	70.3	64.8	72.4	68.8	80.3
Consumed from 4—7 food groups	26.0	24.6	2.9	11.4	22.1	9.7	10.0	10.9	16.1	6.6	11.5
**Meal frequency**											
6 to 8 mo old children	1.8 ± 1.5	1.3 ± 1.5	1.0 ± 1.3	1.8 ± 1.0	1.3 ± 1.5	1.4 ± 1.4	1.7 ± 1.2	1.5 ± 1.5	1.7 ± 1.4	1.0 ± 1.3	2.7 ± 1.7
9 to 11 mo old children	2.0 ± 1.4	1.9 ± 1.7	1.9 ± 1.3	2.2 ± 1.0	1.4 ± 1.6	1.8 ± 1.3	2.0 ± 1.3	2.1 ± 1.4	1.9 ± 1.2	1.5 ± 1.3	2.6 ± 1.7
12 to 23 mo old children	2.2 ± 1.4	2.5 ± 1.6	2.5 ± 1.3	2.3 ± 1.1	1.3 ± 1.7	2.5 ± 1.2	2.3 ± 1.2	2.4 ± 1.5	2.5 ± 1.2	2.3 ± 1.6	3.2 ± 1.7
**Child feeding index**^b^	3.9 ± 1.4	3.7 ± 1.4	3.9 ± 1.0	4.2 ± 1.1	3.6 ± 1.2	3.9 ± 1.2	4.0 ± 1.2	3.9 ± 1.2	4.1 ± 1.2	3.8 ± 1.1	4.0 ± 1.3
	Madagascar	Malawi	Mali	Mozambique	Namibia	Niger	Rwanda	Senegal	South Africa	Zimbabwe	Tanzania
Currently...											
Breastfeeding	85.6	86.2	85.1	83.0	59.1	86.5	93.3	80.3	43.1	68.7	81.3
Uses bottle	2.1	5.6	4.9	77.1	35.4	2.4	5.1	4.8	48.1	7.7	4.4
**Dietary diversity for past 24 h**											
Consumed from no food group	8.8	10.7	25.9	13.7	18.1	22.9	15.6	15.1	7.8	4.4	5.9
Consumed from 1—3 food groups	80.7	69.7	59.5	59.4	57.3	69.7	72.9	63.9	54.0	75.7	77.5
Consumed from 4—7 food groups	10.4	19.6	14.6	26.9	24.6	7.4	11.5	21.0	38.2	19.9	16.6
**Meal frequency**											
6 to 8 mo old children	0.2 ± 0.8	1.6 ± 1.1	1.0 ± 1.2	2.2 ± 1.7	1.7 ± 1.4	1.7 ± 1.6	1.3 ± 1.2	1.2 ± 1.2	1.8 ± 1.3	1.8 ± 1.2	1.9 ± 1.0
9 to 11 mo old children	0.2 ± 0.7	2.1 ± 1.1	1.4 ± 1.1	2.5 ± 1.5	2.0 ± 1.3	2.5 ± 1.7	2.3 ± 1.0	1.6 ± 1.2	2.0 ± 1.1	2.2 ± 1.1	2.2 ± 1.1
12 to 23 mo old children	0.2 ± 0.7	2.2 ± 1.3	2.0 ± 1.1	2.8 ± 1.6	2.5 ± 1.3	3.0 ± 1.7	2.6 ± 0.9	2.2 ± 1.2	2.0 ± 1.0	2.5 ± 1.2	2.4 ± 0.9
**Child feeding index**^b^	3.2 ± 0.8	4.1 ± 1.2	3.8 ± 1.1	3.6 ± 1.3	3.6 ± 1.3	4.2 ± 1.2	4.2 ± 1.1	4.0 ± 1.2	3.3 ± 1.3	4.0 ± 1.1	4.3 ± 1.1

*DRC* Democratic Republic of the Congo

^a^Sample includes only households with complete data on children’s height, age, and haemoglobin levels. Values represent mean ± standard deviation or percentage

^b^Child Feeding Index: A child feeding index was created based on age-specific (6 to 8 months, 9 to 23 months) feeding recommendations on breastfeeding, use of feeding bottle, diet diversity, and meal frequency (Kramer, 2012 [22]; Ruel and Menon, 2002 [17]; WHO, 2010 [21, 24])

### Child feeding practices and its association with nutritional outcome

**Fig. 1:**
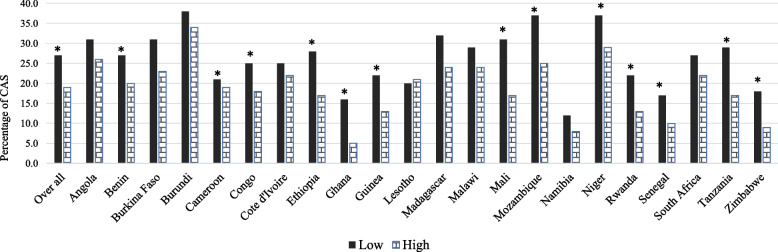
Bars show the observed prevalence of co-occurrence of anemia and stunting (CAS) by child feeding index and country, among children aged 6 to 23 months (*n* = 33,846) in 22 sub-Saharan African countries (pooled data, Demographic and Health Survey data sets, 2008 to 2017). Stars above bars indicate significant difference (*P* < 0.05) in the prevalence of CAS within levels of child feeding scores (i.e., High, Medium, and Low)

Figure 1 shows bivariate associations between feeding practices and CAS for each country. Overall, a lower proportion of children with CAS was associated with having a high compared to a low score on the IYCF score. There were however some country variations in this pattern. After adjusting for other covariates in the pooled data set, child feeding index significantly predicted CAS (See Table 4). That is, overall higher child feeding index score was associated with a lower odds of children being both stunted and anaemic (*p* ≤ 0.001). There were country-specific differences in the models results after adjusting for other covariates (Supplementary Table 1).

**Table 4:** Logistic regression results showing the overall relationship between child feeding index and Childhood anaemia and stunting and their Co-occurrence among children 6 to 23 months^a^

	**Indicator of malnutrition**		
	**Anaemia**	**Stunted**	**CAS**
Child feeding index^b^	1.08***	0.83***	0.86***
	(1.049—1.108)	(0.805—0.848)	(0.836—0.884)
High wealth (Yes)^c^	0.91**	0.76***	0.75***
	(0.833—0.987)	(0.696—0.824)	(0.690—0.824)
Household size (#)	1.04***	0.97***	0.98***
	(1.034—1.054)	(0.960—0.974)	(0.978—0.992)
Household head (Male)	1	0.92*	0.92
	(0.915—1.092)	(0.851—1.004)	(0.837—1.006)
Household head’s education (At least secondary)	0.83***	0.78***	0.76***
	(0.754—0.910)	(0.718—0.849)	(0.696—0.836)
Improved water source for drinking (Yes)^d^	0.99	0.99	1.02
	(0.921—1.069)	(0.930—1.064)	(0.949—1.091)
Improved sanitation (Yes)^e^	0.90***	0.82***	0.81***
	(0.831—0.972)	(0.764—0.885)	(0.745—0.869)
Caregiver’s age (1 = 25 to 49 y; 0 = 15 to 24 y)	0.75***	0.94*	0.87***
	(0.699—0.811)	(0.880—1.006)	(0.813—0.938)
Caregiver’s education (At least secondary)	0.72***	0.73***	0.69***
	(0.656—0.794)	(0.666—0.810)	(0.616—0.767)
Child had diarrhoea in past 2 wk. (Yes)	1.03	1.13***	1.13***
	(0.945—1.116)	(1.047—1.216)	(1.040—1.230)
Child had fever in past 2 wk. (Yes)	1.34***	1.01	1.12***
	(1.230—1.453)	(0.941—1.090)	(1.037—1.213)
Child given iron tablet in past 6 mo (Yes)	1.07	1.01	1.01
	(0.939—1.214)	(0.906—1.130)	(0.898—1.134)
Observations	29,408	29,408	29,408

^a^CAS: Co-occurrence of anaemia (haemoglobin level < 11.0 mmol/L) and stunting (length/height-for-age Z-score < -2). Values are odds ratios (95% confidence intervals) from logistic regression models. Adjusted models are multiple logistic regression with CAS as the dependent variable controlling for all covariates shown; ***p < 0.001, **p < *0.05*

^b^Child Feeding Index: A child feeding index was created based on age-specific (6 to 8 months, 9 to 23 months) feeding recommendations on breastfeeding, use of feeding bottle, diet diversity, and meal frequency (Kramer, 2012 [22]; Ruel and Menon, 2002 [17]; WHO, 2010 [21, 24])

^c^Wealth quintiles were calculated from an asset-based wealth index using assigned asset weights from a principal components analysis to create standardized asset scores

^d^Improved water source: piped household water connection, public standpipe, borehole, protected dug well, protected spring and rainwater

^e^Improved sanitation: ventilated improved pit latrines and pit latrines with a slab or covered pit; NB: There is no data on wealth for South Africa

**Fig. 2:**
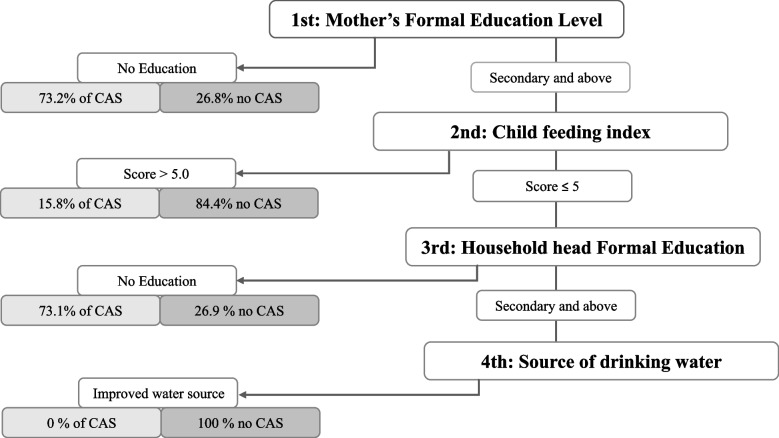
A summary of the decision tree analysis showing the three most important correlates of co-occurrence of anaemia and stunting (CAS) ^1^Improved water source: piped household water connection, public standpipe, borehole, protected dug well, protected spring and rainwater

The decision tree analysis offered an alternative means of identifying the level of relevance of attributes predicting CAS. Overall, the caregiver’s educational level was the most important predictor of CAS, followed by child feeding index score, the household head’s educational level, and access to improved water source for drinking (Fig. 2). A further breakdown of the tree through the nodes revealed that, where caregivers had no education at all or terminated at the primary level, there was 73.1% of CAS and 26.9% of no CAS. However, where the level of education was secondary school and above, the most important predictor towards CAS was the child feeding index score. A feeding index score greater than five resulted in 84.4% chance of no CAS. However, if the child feeding score was less than five, the level of education of the family head was the most important predictor of CAS. If the household head’s level of education was below primary school, this resulted in CAS. However, if the level of education was secondary and above, state of drinking water (i.e., having an improved water source) was the predictor to focus on. Where there was improved water source, there was no CAS, however, all households without improved water source had CAS.

### Other covariates associated CAS

In the pooled analysis, a lower odds of CAS was also associated with greater household wealth, larger family size, higher level of education of the household head and the mother, improved sanitation, and older mothers (See Table 4). Children with recent diarrhoea or fever had a higher odds of CAS. There were country-specific differences in how sociodemographic variables were related to CAS. For example, after controlling for other predictors, more caregiver formal education was associated with a lower odd of a child being both anaemic and stunted only in six countries. Not all countries showed a negative association between wealth and CAS. Improved sanitation was protective against CAS (decreased odds) in Burkina Faso, Burundi, Niger, and Senegal Faso. Having had childhood diarrhoea recently was a predictor of CAS in Angola, Cameroon, and Zimbabwe.

## Discussion

This study aimed to examine the relationship between IYCF recommend practices and the co-occurrence of anaemia and stunting. Arguably, this study provides the most comprehensive up-to-date evidence of the association of IYCF practice on CAS in children 6 to 23 months of age, using nationally representative samples across sub-Saharan African countries. Based on the WHO classification for assessing severity of malnutrition [31], approximately 64% of countries in the current sample had very high prevalence of stunting (≥ 30%). Conversely, the prevalence of childhood anaemia was severe [3] for all the countries in the study (i.e., above 40%). A quarter of the children (25%) were anaemic and stunted at the same time. Overall, the child feeding index was associated with CAS, and the decision tree analysis showed maternal education as the most important predictor of CAS in SSA followed by child feeding index. Result of the influence of child feeding practices on comorbid malnutrition, corroborates with earlier research conducted in other global regional blocks [32, 33]. IYCF score was associated with the decreased likelihood of stunting and CAS, but rather the increased odds of children being anaemic.

The Lancet series on child survival identified IYCF practice as a major factor in children survival [34, 35]. This analysis buttresses the importance of infant and young child feeding practices in child nutritional status and growth. There is a need for continued interventions such as counselling of caregivers about benefits of appropriate or adequate child feeding practices. In the first two years of life, appropriate breastfeeding and complimentary feeding techniques are channels for optimal nutrition that provide defence against poor growth and boost immunity against illnesses.

That IYCF was associated with anaemia but not in the expected direction. This may be because that there are several other factors such as non-iron deficiency (other micronutrients), infections and blood disorders that contributes significantly to anaemia in the sub-Saharan African region. The causes of childhood anaemia are multifaceted and intricately related to one another. There are many non-dietary factors, such as intestinal parasites like soil-transmitted helminths (STH) and Schistosoma; malaria; HIV infection; and chronic diseases like sickle cell disease, in addition to dietary factors (such as iron deficiency, other micronutrient deficiencies such as folate, vitamin B12, and vitamin A), that may be linked to IYCF.

The role mother’s formal education plays in the overall well-being and specifically children’s nutrition and growth has long been documented [36]. Generally, higher educational status of caregivers reflects an improved socioeconomic status that in-turn affects the proximate determinants of health and, either directly or indirectly, affect the health and nutritional status of children [37]. This is however influenced by other socio-environmental factors [38]. An analysis of the effect of parent education on children nutritional status from 56 developing countries, concluded that formal education of the parent might have greater impact on nutrition when the education directly improved nutritional knowledge of parents [39].

Overall, type of water and sanitation were also important predictors of CAS. This finding would suggest support of interventions that addressed environmental enteric dysfunction an underlying cause of both stunting and anaemia, particularly among people living in conditions of poor water, sanitation, and hygiene [40]. However, the SHINE study in Zimbabwe yielded no additional benefit with respect to reduction in stunting and anaemia. The association of sanitation and the presence of fever in children with the CAS reinforces the important role infections play in child growth. Infections causing illnesses such as malaria and diarrhoea result in anaemia and growth faltering in children [41, 42]. Child nutrition and infections have a bidirectional relationship. Whereas frequent infections can lead to diarrhoea and/or impair absorption of needed nutrients thus resulting in malnutrition among children, a malnourished child is also more susceptible to infections. Generally, this study also revealed the disadvantage of poor socio-demographics (via wealth) on the nutrition outcomes, here CAS. This finding is also consistent with previous studies conducted in other global regional blocks [43, 44].

In addition to food security and health care services, the UNICEF conceptual framework considers the importance of immediate determinants such as adequate caregiving practices, including IYCF, to ensure the optimal growth of children. This study's findings demonstrated the impact of IYCF and its importance in a child's development. The effects of some underlining determinations in the framework, such as health (which includes diarrheal status), education, and sanitation, on children's nutritional status are also buttressed in the current study [45].

With respect to policy, countries are urged by the Global Strategy on Infant and Young Child Nutrition to create and put into effect comprehensive IYCF guidelines that safeguard, encourage, and promote breastfeeding and complementary feeding [46]. While efforts to improve child feeding practices among caregivers continue, as a policy recommendation there is an increased need for early childhood educators to be educated on appropriate child feeding practices. This is necessary as we see an increasing number of young children attending day care centres and prekindergarten programs. The WHO and others rightly acknowledge the role these early childhood educators play in optimal infant and child health [47, 48].

A major strength of this work is that the current analysis was drawn from twenty-two nationally representative large samples. Additionally, to our knowledge, this is the first analysis using representative data across SSA to investigate the effect of the WHO’s recommended infant and young children feeding practices on the co-occurrence of stunting and anaemia at the individual level. This notwithstanding, the study is not without some limitations. First, it uses cross-sectional data occurring at different time periods in different countries and that the nutrition outcomes of interest, that is childhood anaemia and stunting could be influenced by prevailing some country-specific microeconomic variables which are not captured in the DHS, thus not included in our current models. Information on the use of micronutrients supplements and/or other drugs by children that could influence their anaemia status in children were not accounted for in the DHS thus could not be controlled for in our models.

Another probable limitation of our estimation technique, particularly in the logistic regression may be that the child feeding index (score) variable may be endogenous to our model, in that it may be influenced by some covariates that also influenced the co-occurrence of stunting and anaemia. The existence of such an issue (endogeneity) could lead to having biased estimates [49]. This concern is often addressed using instrumental variables and a two-stage least squares methods. Thus, future studies could explore identifying a variable that is associated with the endogenous variable being predicted in the first stage equation but not associated with CAS. This may ensure better estimates. Unfortunately, after much exploration we found no such variable or variables in the DHS dataset.

## Conclusions

Although there is no “silver bullet” that could solve the CAS, these results strengthen the argument for the improvement of IYCF and maternal formal education may serve as a pathway to the improvement of childhood nutrition. Interventions aimed at addressing the burden of stunting and anaemia and their co-occurrence are in synchrony with the Sustainable Development Goals aimed at reducing hunger and improving the wellbeing of the populace, particularly women and children.

## Supplementary Information

**Additional file 1: Supplementary Table 1.** Logistic regression results showing the relationship between child feeding index and CAS among children 6 to 23 months by study countries.

## Data Availability

All data generated or analysed during this study are included in this published article [and its supplementary information files].

## References

[1] Subramanian SV, Balarajan Y, Ramakrishnan U, Özaltin E, Shankar AH (2011). Anaemia in low-income and middle-income countries. The Lancet.

[2] Kassebaum NJ, Jasrasaria R, Naghavi M, Wulf SK, Johns N, Lozano R (2014). A systematic analysis of global anemia burden from 1990 to 2010. Blood.

[3] WHO (2011). Haemoglobin concentrations for the diagnosis of anaemia and assessment of severity.

[4] UNICEF (2019). Levels and trends in child malnutrition UNICEF-WHO-World Bank Group joint child malnutrition estimates: Key findings of the.

[5] Oruamabo RS (2015). Child malnutrition and the Millennium Development Goals: Much haste but less speed?. Arch Dis Child.

[6] United Nations. About the Sustainable Development Goals - United Nations Sustainable Development. 2015. Available from: https://www.un.org/sustainabledevelopment/sustainable-development-goals/. [cited 2019 Dec 8].

[7] Walter T (1994). 4 Effect of iron-deficiency anaemia on cognitive skills in infancy and childhood. Baillieres Clin Haematol.

[8] Horton S (2016). Chapter 2.1 The Economics of Poor Nutrition: Patterns, consequences and costs. Good Nutrition: Perspectives for the 21st Century.

[9] Horton S, Ross J (2003). The economics of iron deficiency. Food Policy.

[10] Dewey KG, Begum K (2011). Long-term consequences of stunting in early life. Matern Child Nutr.

[11] Allali S, Brousse V, Sacri AS, Chalumeau M, de Montalembert M (2017). Anemia in children: prevalence, causes, diagnostic work-up, and long-term consequences. Expert Rev Hematol..

[12] Gosdin L, Martorell R, Bartolini RM, Mehta R, Srikantiah S, Young MF (2018). The co-occurrence of anaemia and stunting in young children. Matern Child Nutr.

[13] Mohammed SH, Larijani B, Esmaillzadeh A (2019). Concurrent anemia and stunting in young children: prevalence, dietary and non-dietary associated factors. Nutr J.

[14] Piwoz EG, Huffman SL, Quinn VJ (2003). Promotion and advocacy for improved complementary feeding: Can we apply the lessons learned from breastfeeding?. Food Nutr Bull.

[15] Daelmans B, Mangasaryan N, Martines J, Saadeh R, Casanovas C, Arabi M. Strengthening actions to improve feeding of infants and young children 6 to 23 months of age: Summary of a recent World Health Organization/UN ICEF technical meeting, Geneva, 6–9 October 2008. Food Nutr Bull. 2009;30(2). Available from: www.thelancet.com/journals/lancet/article/PIIS0140-.[cited 2022 Sep 30]. 10.1177/15648265090302S208 20496617

[16] WHO (2016). World health statistics 2016: monitoring health for the SDGs sustainable development goals.

[17] Ruel MT, Menon P (2002). Child feeding practices are associated with child nutritional status in Latin America: innovative uses of the demographic and health surveys. J Nutr.

[18] Boyle EH, King ML, Garcia S, Culver C, Bourdeaux J (2020). Contextual data in IPUMS DHS: physical and social environment variables linked to the Demographic and Health Surveys. Popul Environ.

[19] Vollmer S, Wójcik J. The Long-Term Consequences of the Global 1918 Influenza Pandemic: A Systematic Analysis of 117 IPUMS International Census Data Sets. 2017. Available at SSRN: https://papers.ssrn.com/sol3/papers.cfm?abstract_id=3083584 or 10.2139/ssrn.3083584.

[20] DHS (2020). The DHS Program - Demographic and Health Survey (DHS).

[21] WHO (2010). Anthro for personal computers, version 3.1, 2010: software for assessing growth and development of the world’s children.

[22] Kramer M (2012). Optimal duration of exclusive breastfeeding. Cochrane Database Syst Rev..

[23] WHO J, Organization WH, UNICEF undefined (1981). Joint WHO/UNICEF Meeting on Infant and Young Child Feeding, Geneva 9–12 October 1979: statement, recommendations, list of participants.

[24] WHO (2010). Indicators for assessing infant and young child feeding practices: part 2: measurement.

[25] Kim SS, Rawat R, Mwangi EM, Tesfaye R, Abebe Y, Baker J (2016). Exposure to Large-Scale Social and Behavior Change Communication Interventions Is Associated with Improvements in Infant and Young Child Feeding Practices in Ethiopia. PLoS One..

[26] UNICEF (1990). Strategy for Improved Nutrition of Children and Women in Developing Countries. A UNICEF Policy Review.

[27] Dangour AD, Watson L, Cumming O, Boisson S, Che Y, Velleman Y, et al. Interventions to improve water quality and supply, sanitation and hygiene practices, and their effects on the nutritional status of children. Cochrane Database of Systematic Reviews. 2013;2013(8). Available from: https://www.cochranelibrary.com/cdsr/doi/10.1002/14651858.CD009382.pub2/full. [cited 2022 Dec 20]. 10.1002/14651858.CD009382.pub2 PMC11608819 23904195

[28] Brown KH (2003). Diarrhea and Malnutrition. J Nutr..

[29] Song Y, Psychiatry LYS archives of, 2015 U (2015). Decision tree methods: applications for classification and prediction. Shanghai Arch Psychiatry.

[30] Tong W, Hong H, Fang H, Xie Q, Perkins R (2003). Decision forest: Combining the predictions of multiple independent decision tree models. J Chem Inf Comput Sci.

[31] WHO. Global Database on Child Growth and Malnutrition. 2012 [cited 2019 Oct 20]. Available from: https://www.who.int/nutgrowthdb/about/introduction/en/index5.html.

[32] Hashmi AH, Nyein PB, Pilaseng K, Paw MK, Darakamon MC, Min AM (2019). Feeding practices and risk factors for chronic infant undernutrition among refugees and migrants along the Thailand-Myanmar border: a mixed-methods study. BMC Public Health.

[33] Hipgrave DB, Fu X, Zhou H, Jin Y, Wang X, Chang S (2014). Poor complementary feeding practices and high anaemia prevalence among infants and young children in rural central and western China. Eur J Clin Nutr.

[34] Bryce J, El Arifeen S, Pariyo G, Lanata CF, Gwatkin D, Habicht JP (2003). Reducing child mortality: Can public health deliver?. Lancet..

[35] Black R, Allen L, Bhutta Z, Caulfield L, Lancet MDOT, 2008 U (2008). Maternal and child undernutrition: global and regional exposures and health consequences. The Lancet..

[36] Caldwell JC. Education as a factor in mortality decline an examination of Nigerian data. Population studies. 1979;33(3):395-413.

[37] Makoka D. The impact of maternal education on child nutrition : evidence from Malawi,Tanzania and Zimbabwe. DHS Working Papers; 2013.

[38] Reed BA, Habicht JP, Niameogo C (1996). The Effects of Maternal Education on Child Nutritional Status Depend on Socio-Environmental Conditions. Int J Epidemiol.

[39] Alderman H, Headey DD (2017). How important is parental education for child nutrition?. World Dev.

[40] Humphrey JH, Jones AD, Manges A, Maluccio JA, Mbuya MN, Moulton LH (2015). The sanitation hygiene infant nutrition efficacy (SHINE) trial: rationale, design, and methods. Clin Infect Dis.

[41] Kateera F, Ingabire CM, Hakizimana E, Kalinda P, Mens PF, Grobusch MP (2015). Malaria, anaemia and under-nutrition: Three frequently co-existing conditions among preschool children in rural Rwanda. Malar J.

[42] Danaei G, Andrews KG, Sudfeld CR, Fink G, McCoy DC, Peet E (2016). Risk Factors for Childhood Stunting in 137 Developing Countries: A Comparative Risk Assessment Analysis at Global, Regional, and Country Levels. PLoS Med.

[43] Khan JR, Awan N, Misu F. Determinants of anemia among 6–59 months aged children in Bangladesh: Evidence from nationally representative data. BMC Pediatr. 2016. 10.1186/s12887-015-0536-z PMC4707771 26754288

[44] Singh MB, Fotedar R, Lakshminarayana J, Anand PK (2006). Studies on the nutritional status of children aged 0–5 years in a drought-affected desert area of western Rajasthan. India. Public Health Nutr.

[45] UNICEF Conceptual Framework on Maternal and Child Nutrition - Google Scholar. [cited 2022 Dec 20]. Available from: https://scholar.google.com/scholar?hl=en&as_sdt=0%2C5&q=UNICEF+Conceptual+Framework+on+Maternal+and+Child+Nutrition&btnG=.

[46] WHO G. Global strategy for infant and young child feeding. 2003 [cited 2022 Oct 14]; Available from: https://agris.fao.org/agris-search/search.do?recordID=XF2015038078

[47] World Health Organization. Report of the commission on ending childhood obesity. Geneva: World Health Organization; 2016.

[48] Organization WH. Global strategy for infant and young child feeding. 2003. Available from: https://apps.who.int/iris/bitstream/handle/10665/42590/9241562218.pdf. Cited 1 Oct 2022.

[49] Ruel M, Haddad L (2012). The natural history of growth failure: importance of intrauterine and postnatal periods.

