# Supplementary material for: Infant and young child feeding practices are associated with childhood anaemia and stunting in sub-Saharan Africa
Source: BMC Nutr. 2023 Jan 10;9:9. doi: 10.1186/s40795-022-00667-9 (PMC9832766; doi:10.1186/s40795-022-00667-9)
Supplement: Supplementary file 1 — Additional file 1: Supplementary Table 1. Logistic regression results showing the relationship between child feeding index and CAS among children 6 to 23 months by study countries. [file 40795_2022_667_MOESM1_ESM.docx]

**Supplementary Table 1**: Logistic regression results showing the relationship between child feeding index and CAS among children 6 to 23 months by study countries^1^

|  | **Angola** | **Benin** | **Burkina Faso** | **Burundi** | **Cameroon** | **Cote d'Ivoire** | **DRC** | **Ethiopia** | **Ghana** | **Guinea** |
| --- | --- | --- | --- | --- | --- | --- | --- | --- | --- | --- |
| Child Feeding index^2^ | 0.98 | 0.91 | 0.94 | 0.89** | 0.90 | 0.97 | 0.88** | 0.81*** | 0.71*** | 0.79** |
|  | [0.89, 1.08] | [0.80, 1.03] | [0.85, 1.04] | [0.81, 0.97] | [0.79, 1.01] | [0.83, 1.14] | [0.79, 0.98] | [0.73, 0.89] | [0.59, 0.85] | [0.65, 0.95] |
| High wealth (Yes)^3^ | 0.55** | 1.02 | 0.81 | 0.52*** | 0.45*** | 0.62 | 0.89 | 0.67** | 1.35 | 0.61 |
|  | [0.30, 0.99] | [0.63, 1.65] | [0.60, 1.09] | [0.40, 0.69] | [0.27, 0.75] | [0.38, 1.01] | [0.58, 1.37] | [0.49, 0.91] | [0.72, 2.54] | [0.35, 1.07] |
| Household size (#) | 0.95 | 1.04 | 1.01 | 1.03 | 0.99 | 1.03 | 0.98 | 1.03 | 1.12** | 1.03 |
|  | [0.89, 1.02] | [0.98, 1.10] | [0.98, 1.04] | [0.96, 1.09] | [0.95, 1.03] | [0.99, 1.08] | [0.94, 1.03] | [0.96, 1.10] | [1.02, 1.23] | [0.99, 1.07] |
| Household head (Male) | 1.21 | 1.15 | 0.86 | 0.76 | 0.73 | 0.83 | 0.81 | 1.05 | 2.00** | 1.43 |
|  | [0.77, 1.90] | [0.65, 2.03] | [0.59, 1.27] | [0.56, 1.04] | [0.47, 1.14] | [0.42, 1.65] | [0.57, 1.14] | [0.70, 1.58] | [1.05, 3.80] | [0.82, 2.47] |
| Household head’s education (At least secondary) | 0.88 | 0.75 | 0.47** | 0.73 | 1.18 | 0.84 | 0.59*** | 0.60** | 0.69 | 0.90 |
|  | [0.60, 1.29] | [0.42, 1.33] | [0.26, 0.87] | [0.48, 1.10] | [0.84, 1.65] | [0.46, 1.52] | [0.45, 0.77] | [0.37, 1.00] | [0.38, 1.26] | [0.49, 1.64] |
| Improved water source for drinking (Yes)^4^ | 1.25 | 1.21 | 0.94 | 1.02 | 1.36 | 0.67 | 0.86 | 0.96 | 0.71 | 1.29 |
|  | [0.87, 1.80] | [0.81, 1.79] | [0.74, 1.21] | [0.78, 1.34] | [0.97, 1.90] | [0.41, 1.10] | [0.64, 1.16] | [0.72, 1.27] | [0.41, 1.24] | [0.89, 1.87] |
| Improved sanitation (Yes)^5^ | 0.94 | 1.18 | 0.64** | 0.72*** | 1.37 | 1.12 | 0.84 | 0.82 | 1.62 | 0.65 |
|  | [0.64, 1.38] | [0.67, 2.08] | [0.46, 0.91] | [0.58, 0.90] | [0.93, 2.00] | [0.75, 1.68] | [0.61, 1.15] | [0.49, 1.35] | [0.89, 2.95] | [0.38, 1.10] |
| Caregiver’s age (1= 25 to 49 y; 0=15 to 24 y) | 0.79 | 1.15 | 0.84 | 0.89 | 1.09 | 1.06 | 0.85 | 0.75 | 0.96 | 1.00 |
|  | [0.54, 1.16] | [0.75, 1.76] | [0.65, 1.09] | [0.65, 1.21] | [0.78, 1.51] | [0.64, 1.76] | [0.64, 1.13] | [0.53, 1.06] | [0.51, 1.82] | [0.72, 1.39] |
| Caregiver’s education (At least secondary) | 0.67 | 0.62 | 0.38*** | 0.80 | 0.57** | 0.35 | 0.81 | 0.69 | 0.38*** | 1.05 |
|  | [0.40, 1.11] | [0.31, 1.23] | [0.19, 0.75] | [0.51, 1.25] | [0.37, 0.88] | [0.12, 1.02] | [0.58, 1.13] | [0.34, 1.43] | [0.20, 0.70] | [0.46, 2.41] |
| Child had diarrhoea in past 2 wk (Yes) | 1.98*** | 0.61 | 1.00 | 1.05 | 1.48*** | 1.52 | 0.97 | 1.24 | 1.11 | 0.95 |
|  | [1.34, 2.92] | [0.34, 1.10] | [0.76, 1.32] | [0.82, 1.34] | [1.10, 1.98] | [0.85, 2.71] | [0.65, 1.45] | [0.87, 1.76] | [0.59, 2.10] | [0.58, 1.55] |
| Child had fever in past 1 mo (Yes) | 1.07 | 0.81 | 1.04 | 1.30** | 1.24 | 0.67 | 1.80*** | 1.41 | 0.89 | 0.91 |
|  | [0.73, 1.56] | [0.47, 1.38] | [0.80, 1.35] | [1.02, 1.66] | [0.93, 1.64] | [0.38, 1.17] | [1.35, 2.40] | [0.99, 2.02] | [0.45, 1.77] | [0.60, 1.39] |
| Child given iron tablet in past 6 mo (Yes) | 0.50** | 1.03 | 0.61** | 0.77 | 0.93 | 0.89 | 0.96 | 1.43 | 0.99 | 0.92 |
|  | [0.29, 0.87] | [0.70, 1.52] | [0.39, 0.95] | [0.50, 1.20] | [0.52, 1.68] | [0.52, 1.54] | [0.67, 1.39] | [0.88, 2.34] | [0.56, 1.75] | [0.52, 1.61] |
| Observations | 1,363 | 722 | 197 | 1,747 | 1,539 | 835 | 2,247 | 2,480 | 773 | 910 |

^1^ CAS: Co-occurrence of anaemia (haemoglobin level < 11.0 mmol/L) and stunting (length/height-for-age Z-score < -2). ^2^Child Feeding Index (IYFC): A child feeding index was created based on age-specific (6 to 8 months, 9 to 23 months) feeding recommendations on breastfeeding, use of feeding bottle, diet diversity, and meal frequency (Kramer, 2012; Ruel and Menon, 2002; WHO, 2010); ^3^Wealth quintiles were calculated from an asset-based wealth index using assigned asset weights from a principal components analysis to create standardized asset scores

^4^Improved water source: piped household water connection, public standpipe, borehole, protected dug well, protected spring and rainwater; ^5^Improved sanitation: ventilated improved pit latrines and pit latrines with a slab or covered pit. ****p<0.001, **p<0.05*

**Supplementary Table 1.** Logistic regression results showing the relationship between child feeding index and CAS among children 6 to 23 months^1^

|  | **Lesotho** | **Madagascar** | **Malawi** | **Mali** | **Mozambique** | **Namibia** | **Niger** | **Rwanda** | **Senegal** | **South Africa** | **Zimbabwe** | **Tanzania** |
| --- | --- | --- | --- | --- | --- | --- | --- | --- | --- | --- | --- | --- |
| Child Feeding index^2^ | 1.03 | 0.77*** | 0.90 | 0.87** | 0.80*** | 1.00 | 0.87*** | 0.74*** | 0.82*** | 0.91 | 0.82*** | 0.72*** |
|  | [0.80, 1.33] | [0.65, 0.92] | [0.79, 1.02] | [0.76, 1.00] | [0.72, 0.89] | [0.75, 1.33] | [0.78, 0.96] | [0.63, 0.86] | [0.74, 0.91] | [0.56, 1.47] | [0.72, 0.92] | [0.66, 0.80] |
| High wealth (Yes)^3^ | 0.35** | 1.20 | 0.79 | 0.61** | 0.55*** | 0.32 | 1.14 | 0.74 | 0.37*** |  | 1.34 | 0.68** |
|  | [0.12, 0.97] | [0.81, 1.79] | [0.56, 1.13] | [0.40, 0.93] | [0.39, 0.78] | [0.08, 1.35] | [0.79, 1.64] | [0.49, 1.12] | [0.25, 0.56] |  | [0.80, 2.22] | [0.50, 0.92] |
| Household size (#) | 1.02 | 1.05* | 1.02 | 1.04* | 1.02 | 0.95 | 1.03 | 1.02 | 1.00 | 1.27* | 0.96 | 1.01 |
|  | [0.89, 1.16] | [1.00, 1.11] | [0.95, 1.10] | [1.00, 1.08] | [0.96, 1.08] | [0.84, 1.07] | [0.99, 1.07] | [0.92, 1.13] | [0.99, 1.01] | [0.99, 1.64] | [0.89, 1.04] | [0.98, 1.03] |
| Household head (Male) | 0.93 | 1.23 | 1.01 | 0.83 | 1.04 | 1.08 | 1.08 | 0.91 | 0.93 | 0.21** | 0.97 | 1.18 |
|  | [0.45, 1.93] | [0.81, 1.88] | [0.66, 1.53] | [0.48, 1.43] | [0.76, 1.43] | [0.43, 2.68] | [0.69, 1.70] | [0.55, 1.49] | [0.68, 1.27] | [0.05, 0.97] | [0.62, 1.51] | [0.83, 1.68] |
| Household head’s education (At least secondary) | 0.74 | 0.89 | 0.70** | 0.39*** | 0.79 | 1.02 | 0.46** | 0.68 | 0.88 | 0.92 | 1.06 | 0.95 |
|  | [0.37, 1.49] | [0.56, 1.41] | [0.50, 0.99] | [0.19, 0.78] | [0.51, 1.22] | [0.32, 3.24] | [0.24, 0.89] | [0.32, 1.45] | [0.56, 1.37] | [0.19, 4.39] | [0.65, 1.72] | [0.66, 1.36] |
| Improved water source for drinking (Yes)^4^ | 1.02 | 1.33 | 0.87 | 1.16 | 1.19 | 1.82 | 0.92 | 1.26 | 1.18 | 1.27 | 0.93 | 1.11 |
|  | [0.51, 2.04] | [0.88, 2.03] | [0.59, 1.28] | [0.86, 1.57] | [0.90, 1.59] | [0.75, 4.45] | [0.69, 1.25] | [0.87, 1.82] | [0.93, 1.50] | [0.28, 5.76] | [0.62, 1.38] | [0.89, 1.38] |
| Improved sanitation (Yes)^5^ | 1.18 | 1.13 | 1.08 | 0.85 | 0.96 | 1.9 | 0.54** | 0.87 | 0.71*** | 0.24 | 1.01 | 0.85 |
|  | [0.62, 2.23] | [0.55, 2.28] | [0.75, 1.54] | [0.59, 1.21] | [0.65, 1.43] | [0.63, 5.73] | [0.34, 0.87] | [0.60, 1.27] | [0.55, 0.92] | [0.05, 1.13] | [0.68, 1.49] | [0.67, 1.08] |
| Caregiver’s age (1= 25 to 49 y; 0=15 to 24 y) | 0.63 | 1.01 | 0.61*** | 0.81 | 0.62*** | 0.64 | 0.87 | 1.06 | 1.08 | 0.42 | 0.88 | 0.90 |
|  | [0.36, 1.10] | [0.74, 1.38] | [0.43, 0.88] | [0.59, 1.11] | [0.47, 0.82] | [0.21, 1.94] | [0.63, 1.19] | [0.66, 1.70] | [0.85, 1.37] | [0.08, 2.17] | [0.58, 1.35] | [0.70, 1.15] |
| Caregiver’s education (At least secondary) | 1.00 | 0.97 | 1.11 | 0.94 | 0.72 | 0.78 | 0.43** | 0.68 | 1.01 | 0.13*** | 0.61** | 0.93 |
|  | [0.47, 2.12] | [0.57, 1.64] | [0.74, 1.66] | [0.45, 1.94] | [0.44, 1.17] | [0.23, 2.61] | [0.20, 0.93] | [0.35, 1.29] | [0.66, 1.53] | [0.03, 0.60] | [0.40, 0.95] | [0.63, 1.36] |
| Child had diarrhoea in past 2 wk (Yes) | 0.9 | 0.92 | 1.23 | 0.99 | 1.28 | 0.89 | 1.18 | 1.42 | 0.82 | 0.02 | 1.47** | 0.93 |
|  | [0.44, 1.85] | [0.59, 1.43] | [0.92, 1.66] | [0.64, 1.53] | [0.88, 1.87] | [0.32, 2.46] | [0.86, 1.62] | [0.93, 2.17] | [0.63, 1.08] | [0.00, 2.37] | [1.03, 2.09] | [0.71, 1.21] |
| Child had fever in past 1 mo (Yes) | 0.69 | 1.42* | 0.88 | 1.32 | 1.08 | 0.90 | 0.59*** | 0.72 | 1.02 | 0.58 | 0.74 | 1.32** |
|  | [0.35, 1.37] | [0.99, 2.04] | [0.65, 1.19] | [0.86, 2.01] | [0.77, 1.52] | [0.29, 2.81] | [0.41, 0.85] | [0.46, 1.12] | [0.77, 1.34] | [0.06, 5.71] | [0.43, 1.26] | [1.01, 1.73] |
| Child given iron tablet in past 6 mo (Yes) |  | 1.64 | 0.76 | 1.03 | 1.00 |  | 0.92 |  | 1.36 |  |  | 1.88 |
|  |  | [0.57, 4.72] | [0.46, 1.27] | [0.70, 1.52] | [0.69, 1.45] |  | [0.57, 1.48] |  | [0.81, 2.28] |  |  | [0.81, 4.35] |
| Observations | 372 | 1,210 | 1,305 | 1,260 | 1,413 | 267 | 1,273 | 999 | 2,960 | 89 | 1,207 | 2,558 |

^1^ CAS: Co-occurrence of anaemia (haemoglobin level < 11.0 mmol/L) and stunting (length/height-for-age Z-score < -2). Values are odds ratios (95% confidence intervals) from logistic regression models. Adjusted models are multiple logistic regression with CAS as the dependent variable controlling for all covariates shown; ****p<0.001, **p<0.05,* ^2^Child Feeding Index: A child feeding index was created based on age-specific (6 to 8 months, 9 to 23 months) feeding recommendations on breastfeeding, use of feeding bottle, diet diversity, and meal frequency (Kramer, 2012; Ruel and Menon, 2002; WHO, 2010); ^3^Wealth quintiles were calculated from an asset-based wealth index using assigned asset weights from a principal components analysis to create standardized asset scores

^4^Improved water source: piped household water connection, public standpipe, borehole, protected dug well, protected spring and rainwater; ^5^Improved sanitation: ventilated improved pit latrines and pit latrines with a slab or covered pit.
